# Successful treatment of necrotising fasciitis following cobra bite: a case report

**DOI:** 10.1093/jscr/rjaf309

**Published:** 2025-05-20

**Authors:** Santosh Dev, Amit Yadav, Prajjwol Luitel, Barsha Dev, Gyaneshwor Shrestha, Badal Karki

**Affiliations:** Department of General Surgery, Tribhuvan University Teaching Hospital, Maharajgunj Road, Kathmandu 44600, Nepal; Maharajgunj Medical Campus, Institute of Medicine, Tribhuvan University, Maharajgunj Road, Kathmandu 44600, Nepal; Maharajgunj Medical Campus, Institute of Medicine, Tribhuvan University, Maharajgunj Road, Kathmandu 44600, Nepal; Nepalgunj Medical College Teaching Hospital, Nepalgunj, Banke, Kohalpur 21904, Nepal; Department of General Surgery, Tribhuvan University Teaching Hospital, Maharajgunj Road, Kathmandu 44600, Nepal; Department of General Surgery, Tribhuvan University Teaching Hospital, Maharajgunj Road, Kathmandu 44600, Nepal

**Keywords:** cobra bite, necrotising fasciitis, debridement, skin graft

## Abstract

Necrotising fasciitis following cobra envenomation is an infrequent but life-threatening complication. We report a case of a 72-year-old woman from Khotang who developed a rapidly progressive, discharging wound on her right foot 3 days after sustaining a cobra bite. Initially treated at a local snakebite center with 30 vials of antivenom, she later presented with tachycardia, low-grade fever, and leukocytosis. Local tissue culture revealed *Streptococcus pyogenes*, prompting escalation of antibiotics to Piperacillin-Tazobactam. The ensuing extensive tissue necrosis, driven by venom cytotoxicity, facilitated secondary bacterial colonization. Aggressive resuscitation, repeated surgical debridement, and nutritional support with a high-protein diet and multivitamins resulted in robust granulation tissue formation. Definitive reconstruction was achieved with a split-thickness skin graft. This case underscores that while antivenom reverses systemic toxicity, early recognition and prompt surgical intervention are paramount in preventing the devastating progression of local necrosis to necrotising fasciitis.

## Introduction

Wound infections following snakebites including cellulitis, abscess formation, tissue necrosis, gangrene, and necrotising fasciitis have been documented in the literature, with an overall prevalence of ⁓27% (95% CI: 22.0%–32.0%) [1–3]. Among these complications, necrotising fasciitis is notably rare, with only a limited number of cases reported [4, 5]. This condition is characterized by a rapidly progressive infection that destroys the fascia and subcutaneous tissue, leading to extensive necrosis [6]. Snakebite remains a critical yet often overlooked global health issue, with 20 000–40 000 cases and up to 3000 deaths annually in Nepal, figures that likely underestimate the true burden due to underreporting [7, 8]. About 89 snake species have been recorded in Nepal, among which 17 are venomous. These snakes can be subdivided further into two groups: Elapidae (kraits, cobras, king cobras, and coral snakes) and Viperidae (true viper and pit viper) [9].

The clinical effects of envenomation vary depending on the toxin profile of the snake venom, which may be cytotoxic, neurotoxic, hemotoxic, myotoxic, or cardiotoxic [10]. Importantly, the cytotoxic effects of the venom can cause substantial local tissue destruction and devitalization, thereby predisposing the wound to secondary bacterial infections, which, if not treated promptly, may progress to life-threatening conditions such as necrotising fasciitis [2].

Following the Surgical CAse REport (SCARE) guidelines 2023, we present the successful management of a case of necrotising fasciitis secondary to a cobra bite, emphasizing early surgical intervention, nutritional support, and awareness of local complications beyond systemic envenomation [11].

## Case presentation

A 72-year-old woman without prior comorbidities from Khotang district presented to emergency department with a rapidly enlarging, discharging wound on her right foot. She had sustained a cobra bite 3 days earlier while collecting biomass fuel in her garden. Initially, she experienced localized pain and swelling at the bite site. Shortly after the incident, she was taken to a local snakebite treatment center. There, she was admitted to the intensive care unit, intubated for 2 days, and received 30 vials of polyvalent antivenom which is effective against the four common species of snakes found in India; Russell's Viper (*Daboia russelii*), Common Cobra (*Naja naja*), Common Krait (*Bungarus caeruleus*), and Saw Scaled Viper (*Echis carinatus*). Although she was extubated and transferred to the general ward on the third day, she subsequently noted the development of a non-healing wound on her right foot despite routine dressings.

**Figure 1:**
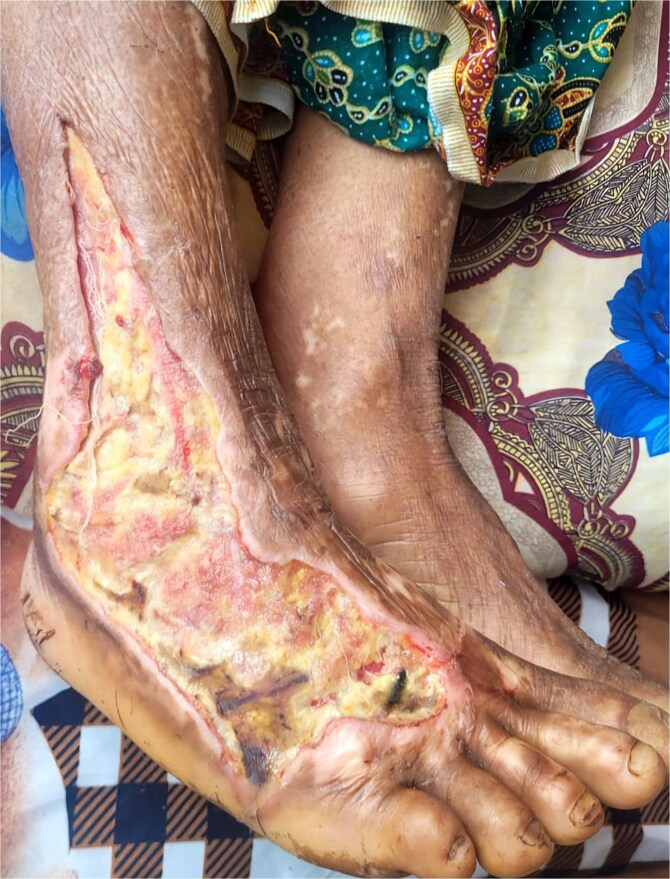
Defect showing extensive tissue damage on the right foot extending to the lower leg.

Upon arrival at our facility on day three post-bite, vital signs were normal except for tachycardia (115 beats per minute) and a low-grade fever (100°F). Local examination revealed a 20 × 9 cm lesion that extended from the right foot to the lower leg (Fig. 1). Laboratory investigations revealed an elevated total leukocyte count of 21 000 cells/mm^3^, a hemoglobin level of 10 mg/dL and a serum albumin of 3.4 g/dL Renal function tests, liver function tests, bleeding time and clotting time were normal. Based on these, she was diagnosed with necrotizing fasciitis type II.

Prompt resuscitation was initiated, empirical antibiotics were started followed by emergency surgical debridement. A culture of the necrotic tissue isolated *Streptococcus pyogenes*, prompting an upgrade in antibiotic therapy to Piperacillin-Tazobactam according to the culture sensitivity results.

**Figure 2:**
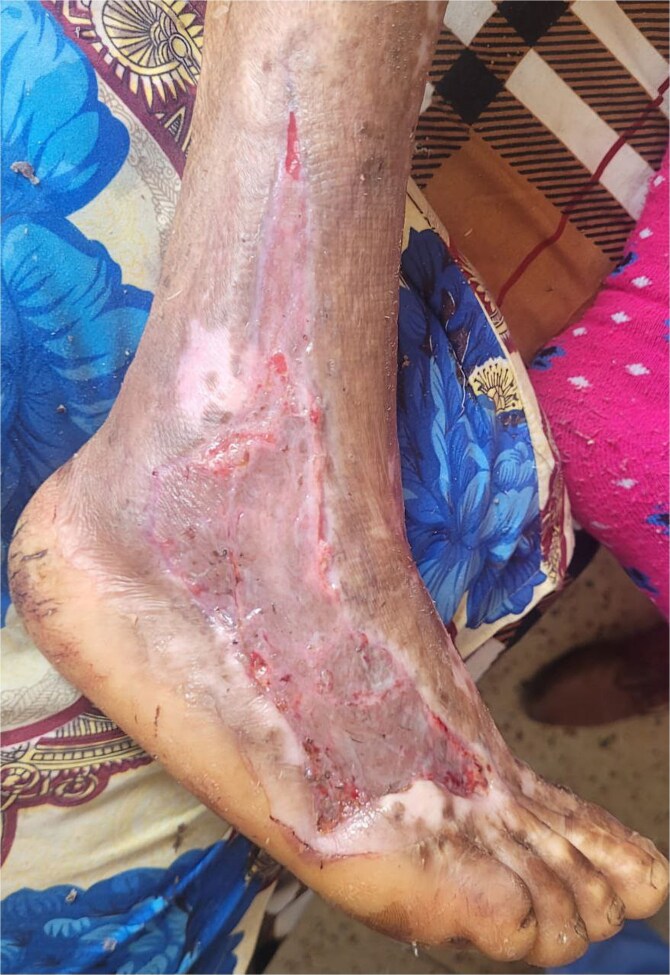
Successful post-operative outcome following split-thickness skin grafting, with complete wound closure and restored skin integrity.

She underwent repeated debridement and daily wound dressings with normal saline, under antibiotic coverage. A high-protein diet (⁓4–5 eggs per day) and multivitamin supplementation were started for nutritional support and to promote wound healing. Over the subsequent two weeks, the wound exhibited good granulation tissue formation, allowing for definitive surgical reconstruction via a split-thickness skin graft harvested from the left thigh (Fig. 2). The patient’s postoperative course was uneventful, and she was discharged in stable condition on day 15, with significant wound closure and improved overall status.

## Discussion

Although necrotising fasciitis is an uncommon complication of snakebites, it has been reported with cobra envenomation in India, Nigeria, Taiwan, Vietnam, and Namibia [2, 5]. In Nepal, while cobra bites are often recognized for their neurotoxic effects, they can also induce severe local tissue damage via cytotoxins such as phospholipase A2, phosphodiesterases, hyaluronidases, peptidases, and metalloproteinases [9]. Higher doses of cytotoxic venom led to more extensive tissue necrosis, which in turn creates a favorable environment for opportunistic colonization by bacteria from the snake’s oral flora, the patient’s skin, or the external environment [2]. In our case, requiring 30 vials of antivenom suggested substantial envenomation and tissue destruction, culminating in necrotising fasciitis. Microbiological studies of snakebite-related infections often isolate *Morganella morganii*, *Enterococcus* spp., and *Staphylococcus aureus* [1]. However, our culture yielded *S. pyogenes*, possibly reflecting regional bacterial flora differences.

Early cobra bite symptoms include localized pain, swelling, erythema, and warmth, which can quickly progress to skin breakdown, gangrene, or non-healing ulcers—sometimes within hours [5, 7]. In advanced stages, patients may present with fever, tachycardia, and sepsis [6]. Our patient arrived on day three with a discharging wound extending from the right foot to the lower leg, accompanied by fever, tachycardia, and leukocytosis. This clinical picture aligns with the literature, which states that while antivenom neutralizes systemic toxicity, it does not reverse established local necrosis [12].

Necrotising fasciitis should be suspected in any patient with severe soft tissue infection, especially when systemic signs like fever or hemodynamic instability are evident. Key indicators include crepitus, rapid deterioration, and pain that surpasses visible skin findings. Early surgical debridement significantly improves outcomes, with the best survival rates seen when intervention occurs within 24 h, and even better if performed within six [13, 14]. In our case, the patient received prompt management at a district hospital with plastic surgery facilities, allowing for timely debridement and grafting. This crucial mantra has been highlighted in prior case where a 2.5 year male succumbed to death due to failure to recognize the necrotizing fasciitis in time [15].

Management depends on a multifaceted approach: antibiotic therapy, resuscitation, and vigilant critical care. However, urgent surgical intervention—encompassing emergency fasciotomy and repeated debridement—remains paramount to eliminate necrotic tissue and control infection [5, 14]. Nutritional support is vital once the patient is hemodynamically stable, and split-thickness skin grafting can restore wound integrity once granulation tissue forms [6]. In our patient, antibiotics coverage (Piperacillin-Tazobactam) guided by culture results, alongside a high-protein diet and multivitamins, facilitated healing. Consequently, she underwent successful grafting and was discharged in stable condition on postoperative day 15.

## Conclusion

Our case highlights successful and definitive treatment for Necrotising fasciitis of the foot resulting from a cobra bite with early and regular debridement, dressing, with appropriate antibiotics and closure of defect with skin graft. When dealing with Nepalese cobra bites, high index of suspicion should be kept for rapidly enlarging, painful wound following cobra bite in tropical areas as early diagnosis and treatment has favorable outcome.

## Data Availability

The datasets used during this study will be available from the corresponding author upon reasonable request.
